# An Approach to an Inhibition Electronic Tongue to Detect On-Line Organophosphorus Insecticides Using a Computer Controlled Multi-Commuted Flow System

**DOI:** 10.3390/s110403791

**Published:** 2011-03-28

**Authors:** Gustavo A. Alonso, Rocio B. Dominguez, Jean-Louis Marty, Roberto Muñoz

**Affiliations:** 1 Centro de Investigación y de Estudios Avanzados del Intituto Politecnico Nacional, Av. Instituto Politécnico Nacional 2508, Mexico; E-Mails: rdominguez@cinvestav.mx (R.B.D.); rmunoz@cinvestav.mx (R.M.); 2 Université de Perpignan Via Domitia, IMAGES EA4218, Centre de Phytopharmacie, 52 Avenue Paul Alduy, 66860 Perpignan Cedex, France; E-Mail: jlmarty@univ-perp.fr

**Keywords:** biosensor, enzyme inhibition, flow system, multi-commuted, organo-phosphorus insecticides, potentiostat

## Abstract

An approach to an inhibition bioelectronic tongue is presented. The work is focused on development of an automated flow system to carry out experimental assays, a custom potentiostat to measure the response from an enzymatic biosensor, and an inhibition protocol which allows on-line detections. A Multi-commuted Flow Analysis system (MCFA) was selected and developed to carry out assays with an improved inhibition method to detect the insecticides chlorpyrifos oxon (CPO), chlorfenvinfos (CFV) and azinphos methyl-oxon (AZMO). The system manifold comprised a peristaltic pump, a set of seven electronic valves controlled by a personal computer electronic interface and software based on LabView® to control the sample dilutions into the cell. The inhibition method consists in the injection of the insecticide when the enzyme activity has reached the plateau of the current; with this method the incubation time is avoided. A potentiostat was developed to measure the response from the enzymatic biosensor. Low limits of detection of 10 nM for CPO, CFV, and AZMO were achieved.

## 1. Introduction

Automated flow techniques have had a profound impact on how modern analytical procedures are implemented. The concept of Flow Injection Analysis (FIA) was firstly proposed by Ruzicka and Hasen as a new automated liquid handling technique to perform wet chemistry [1]. In the FIA technique, a well defined sample is injected into a continuous carrier stream within a unique manifold. The final chemical species is formed by dispersion and can be measured by coupling a suitable detector [2]. Due to its inherent disadvantages, such as high reagent consumption and a non-reconfigurable manifold to perform multireagent analysis, a new technique named Sequential Injection Analysis (SIA) was introduced in 1990 by Ruzicka and Marshall [3]. In the SIA technique, several reagents can be sequentially aspirated by a multiport valve and stored in a holding coil. Then the flow is stopped and changed, and reagents are propelled to the final reactor (mixing chamber, coil *etc.*). The final chemical species can be measured with any suitable detector [4]. Nowadays, both techniques have been applied for handling liquid solutions and, consequently, performing methods related to wet chemical analysis [5]. However, in recent years, several changes have been introduced in order to increase the degrees of automation and miniaturization, reduce reagent consumption, improve repeatability and generate the lowest possible wastes [6].

One of those changes is the incorporation of solenoid valves into the manifold. Some years ago, Turyan employed a set of magnetic snap valves coupled with a pump to design a new instrumental implementation of a SIA system, named SISA [7]. The manifold had six equivalent inlet/outlet channels; each channel was individually closed or opened by actuating the valve via software. A combination of pumping direction and opening or closing the valves allowed the user to perform different operations. The detection was made with stripping voltammetry. Recently, a hybrid technique, named multi-commuted flow analysis (MCFA) has been successfully tested in automated analytical methods for insecticide detection [6,8]. This technique allows the user to obtain several manifolds without any physical reconfiguration, just by coupling a set of software controlled solenoid valves with a peristaltic pump.

The described flow systems are coupled with a wide variety of detectors [9]; however electrochemical methods such as potentiometry and amperometry are particularly interesting because of their low cost, small size and the possibility of performing *in situ* measurements. Custom electrochemical instrumentation has been developed by several laboratories.

Many researchers have developed single chip potentiostats reducing chip size and cost. Fidler [10] designed a potentiostat based on voltage-controlled current source for an amperometric gas sensor. Turner [11] presented a basic CMOS integrated potentiostat, Kakerow [12] used a monolithic potentiostat, Bandyopadhyay [13] proposed a multi-channel potentiostat, and Frey [14] reported an integrated potentiostat for biosensor chips. New trends in potentiostats are focused in their portability and *in situ* use [15,16]. As a result, the measurement of the screen-printed enzymatic response by an automatic system using the proposed potentiostat can be carried out in daily life [15].

In the present work we propose an automatic online determination system integrated by a set of seven independent solenoid valves coupled with a peristaltic pump, a potentiostat controlled via software which is implemented and calibrated for insecticide detection. The organophosphorus insecticides used in the present work are in the list of priority substances in the field of water policy of the European Community [17]. The full system works as an automatic batch instrument, the peristaltic pump and solenoid-valves are programmed by an interface which can performance several tasks to complete the process of enzymatic inhibition. The performance of the potentiostat designed and assembled in our laboratory was tested according to the sensitivity, accuracy to generate and acquire analog signals, and power-consumption. The improved inhibition protocol allows on-line detection of organophosphorus insecticides by directly measuring the slope of the sensor response.

## 2. Experimental

### 2.1. Reagents

Genetically-modified AChE from Drosophila B131 was produced by Protein Bio Sensor (PBS, Toulouse, France). Acetylthiocholine chloride (ATChCl) and 5,5′-dithiobis(2′-nitrobenzoic acid) (DTNB) were supplied by Sigma-Aldrich (Switzerland). Stock solutions of the enzymes were prepared in 0.1 M phosphate buffer (Na_2_HPO_4_/Kh_2_PO_4_, Sigma-Aldrich, Switzerland) at pH 7.0. Chlorpyrifos-oxon, chlorfenvinphos and azinphos methyl-oxon were purchased from Ehrenstorfer (Augsburg, Germany). Stock solution of insecticides (0.1 m) was prepared in acetonitrile (Carlo Erba Reagenti, Italy) and working insecticide solutions were prepared daily in distilled water by dilution from the stock solutions. Silver (Electrodag PF 410), graphite (Electrodag 423 SS) and silver/silver chloride (electrodag 418 SS) links were purchased from Acheson (Plymouth, UK). Cobalt-phtalocyanine-modified carbon paste was purchased from Gwent Electronic Materials, Ltd (Gwen, UK). Polyvinylchloride (PVC) sheets (200 × 100 mm, 0.5 mm thickness) used as support for the electrodes were obtained from SKK (Denzlingen, Germany). The photocrosslinkable polymer polyvinyl alcohol PVA-AWP (Toyo, Japan) was used for immobilization of enzymes.

The screen-printed three-electrode system with cobalt-phthalocyanine-modified carbon as working electrode, graphite as counter electrode and Ag/AgCl as a reference electrode were fabricated using a DEK248 screen-printing system (Weymounth, UK). Data acquisition software developed in LabView 8.5v (NI, USA).

### 2.2. Biosensor Construction

The biosensor preparation was based on the same protocol used in previous reported works [18–22] which is briefly as follows: the three channel (reference, working and auxiliary electrodes) biosensors were prepared on the PVC support by a screening technique with a DEK 248 printing machine. The following layers were successively printed: a carbon layer, a reference electrode consisting of Ag/AgCl paste printed over the carbon layer in one of the channels, the working electrode, containing the chemical mediator cobalt-phthalocyanine-modified carbon paste, and an insulating layer, prepared in order to maintain uncovered only at 9 mm long portion of the silver track, necessary for the electrical contact. An enzymatic solution (3 μL) containing 30% of enzyme solution (1.12 UI/mL measured by Ellman method [23]) and 70% PVA-AWP were deposited manually on the surface of the working electrode. The biosensors were exposed under neon light at 4 °C for 3 h. Finally the biosensors were held for about three days at room temperature before use. The substrate calibration curves of the biosensor used in the present work have been described in [24].

### 2.3. Flow System Manifold

**Figure 1.**
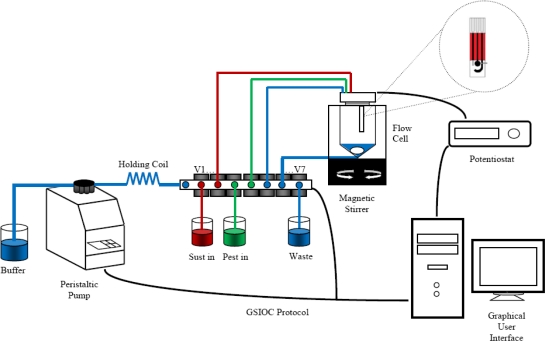
Manifold of the designed MCFA system.

The multi-Commuted Flow Analysis (MCFA) scheme is shown in Figure 1. The complete system was constructed with the following components: a Minipulse3 peristaltic pump (Gilson, France), seven 12VDC 30 PSI pinch valves (NResearch, USA), driven by an ULN2003 (ST, Microelectronics, USA), a knotted reactor made from PTFE tube of 20 cm × 1 mm (L × i.d.), one potentiostat developed in our laboratory with a sensitivity of 1 nA. The potentiostat applies a constant voltage to the electrode and the recorded current is sent to a 2 GHz Pentium IV PC windows XP running LabView8.5 where a control program was implemented.

### 2.4. Potentiostat Circuit Description

Basically, a potentiostat is an electronic device that controls the voltage difference between a working electrode and a reference electrode [16]. In other words, the potentiostat has two tasks. Firstly, it measures the potential difference between a working electrode and reference electrode without polarizing the reference electrode, and compares the potential difference with a preset voltage. Secondly, it injects a current flowing from a counter electrode to a working electrode in order to counter-act the difference between the preset voltage and the existing working electrode potential [25].

**Figure 2.**
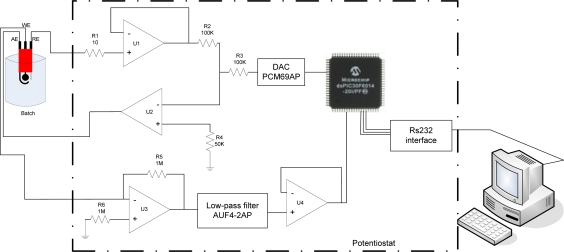
The circuit diagram of the potentiostat.

Figure 2 shows the circuit diagram of a portable potentiostat. The proposed potentiostat is mainly constructed by a dsPIC30F6014 microcontroller, a digital/analog converter, a current to voltage converter, an inverter amplifier, one low-pass active filter and a RS232 serial data hardware interface.

In this potentiostat, the preset voltage (100 mV) is set with the microcontroller and applied by the digital/analog converter (PCM67P/U), the potential of the cell is measured by the U1 and compared by U2 applying the difference of voltage to the cell.

The working electrode is connected at the inverter terminal of a current-voltage converter U3, and is thus held at a potential near to analog ground. Current flowing into the working electrode is converter in output voltage by the amplifier U3. This voltage is a rate proportional to the magnitude of the working electrode current giving for Equation (1). The output of U3 (V_U3_) is connected into a low-pass active filter and after to an inverter U4. The analog signal is acquired by the Analog/Digital converter peripheral of the dsPIC30F6014 microcontroller which sends the digital data to a PC by RS232 interface:

(1) $$V_{U3}=I_{\mathrm{WE}}\mathrm{xR}5$$

### 2.5. Software

The software was developed based on Labview® and the programmed commands control the pump, valves, and the start of acquisition of the experiment. All the elements are synchronized by a visual interface. To perform the experimental assays the program executes a sequence of pre-defined orders which complete a task. In fact, every task is a combination of orders such as pump speed, flow direction and valves opening and closing. The SIA system carries out five principal automatic tasks, set-up of the system; injection of 10 mL of buffer to the cell, injection of 100 μL of substrate, injection of 100 μL of insecticide and drain. The acquisition step starts when the substrate is added and finishes when a slope of inhibition is reached.

### 2.6. Inhibition Protocol

The present work is focused on the determination of the affinity of AChE towards three pesticides. This is expressed by the irreversible inhibition constants (k*_p_*) [26,27] measured with an immobilized enzyme.

**Figure 3.**
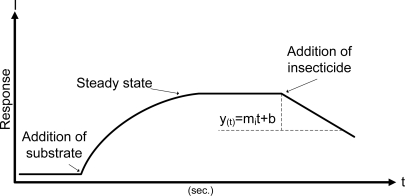
Protocol of enzyme inhibition in the steady-state to measure the inhibition response. The *k_p_* rate is determined by measuring the slope *m_i_*.

The values of kp are calculated by on-line measurements of the rate of inhibition. The amperometric measurements were carried out as follows. The biosensor was vertically inserted into an analytical cell, 10 mL of phosphate buffer was injected at 9.9308 mL/min, under constant magnetic stirring (174 rpm) at constant temperature (30 °C). Then ATChCl solution (final concentration in the cell 1 mM) was injected at 923 μL/min and the signal (steady-state current) by the enzymatic reaction was recorded. This step was repeated to ensure the stability of the biosensor. Finally in the steady-state step a known concentration of insecticide was injected in the same solution at 923 μL/min and a decrease of the current was analyzed Figure 3.

The concentration of insecticide is correlated with the value of slope measured. The protocol presented in this work is classified as *a single step*, according with Arduini *et al.* [28]. The analysis of the inhibition data was computed 2.5 min after of injection of pesticide, with an acquisition rate of 1 sample/s. 150 points per assay were analyzed.

The modeling is determined by the function *polyfit* which finds the coefficients of a polynomial *p*(*t*) of degree *n*, that fits a set of experimental data, *p*(*t*) to *y*(*x*) where *p*(*t*) is the polynomial established see Equation (2) and *y*(*x*) are the experimental data. The analysis is carried out by linear least squares which gave a better fitted regression than linear regression analysis. The function *polyfit* is programmed in LabView 8.5 ®. *Polyfi*t returns value of the slope and interception point:

(2) $$p_{\left(t\right)}=m_{i}t+b$$

## 3. Results and Discussion

**Figure 4.**
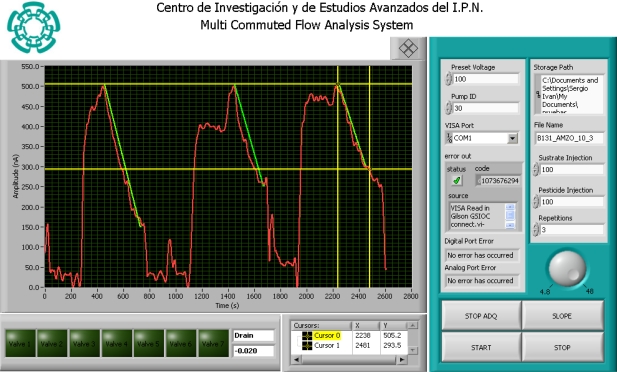
Protocol of enzyme inhibition in the steady-state to measure the inhibition response.

The front panel is shown in Figure 4. The first right side section controls the physical hardware in the MCFA manifold (pump, valves and acquisition); the indicators show the status of every device and alert of any undesirable behavior. In addition, there are controls to customize the experimentats (number of experiments, injected sample, preset voltage and storage options such as file name and path). The acquired signals and the valve status are shown in the left part of the front panel. With the cursors, a specific portion of the acquired signal can be selected for calculating the slope. The current system task and the calculated slope are shown next to the valves indicators.

In order to test the performance of the potentiostat, three aspects were evaluated. The accuracy of the patterns generated and acquired signals, limit of detection, and power-consumption.

**Figure 5.**
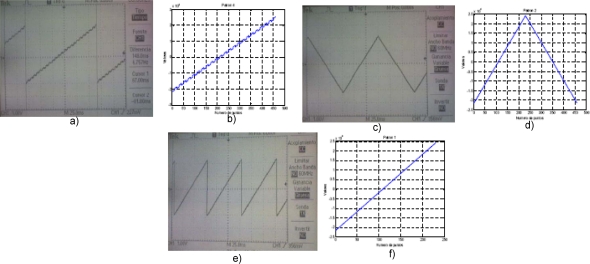
Generated patterns. **(a)**, **(c)**, **(e)** correspond to the analog patterns (obtained). **(b)**, **(d)** and **(f)** correspond to computed patterns (expected).

Figure 5 shows three different patterns generated with the microcontroller dsPIC and converted by the DAC PCM69AP. The patterns were computed first in LabView®, after in the dsPIC30F6014 a program with the commands to generate the patterns were computed; finally the output voltage from PCM69AP was registered by the ADC of the microcontroller and shown in an interface.

The sensitivity of the potentiostat was measured using a dummy cell [25], with different resistive loads to generate different impedances in the cell with the goal to obtain different working electrode currents. The detection limit of current was 2 nA. The power consumption was determined experimentally as 914.89 μA, with a Vdd to Vss potential difference of 5 V. This yields a calculated power of 4.57 mW.

The characterization of the MCFA system was performed using it for measuring different automatically volume injections to obtain dispersion of the data at two injection speeds. To check the reproducibility of the system, different volumes were prepared by the system and the mean values were calculated for two rates. Giving as result a higher precision for small volumes with slow rates, for large volumes the value of the rate can be higher with good accuracy, the repetitivity ranged from 1.48–1.88% RSD. See Table 1.

As a final test, the complete system was used to detect three organophosphorus insecticides: chlorpyrifos-Oxon (CPO), chlorfenvinfos (CFV), azinphos methyl-oxon (AZMO) using a screen-printed enzymatic biosensor based on genetically-modified B131enzyme.

MCFA system carries out a single inhibition in five tasks: first task, the set-up and the injection of 10 mL of phosphate buffer pH = 7 at 48 rpm; second task the injection of 100 μL of substrate ATCh-Cl at 4.8 rpm, with the aim of testing the stability of the biosensor, tasks 1, and 2, are repeated two times. Third task, injection of 100 μL of one preset concentration of insecticide; fourth task calculation of the slope of inhibition; fifth task drain and wash the cell. Three preset concentrations were chosen (10 nM, 1 μM, 0.4 mM) for each insecticide. Each concentration was repeated three times.

**Figure 6.**
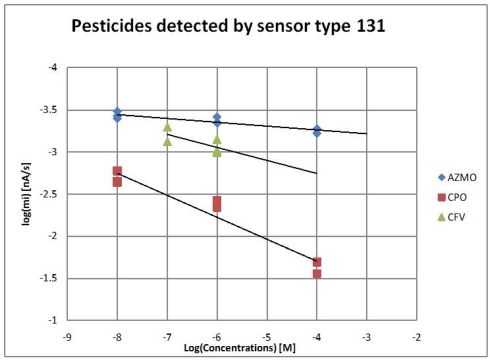
Inhibition response to CPO, CFV and AZMO. Logarithmic dependence of inhibition response.

The values of *m_i_* computed from single inhibitions for three insecticides are shown in Figure 6. With the inhibition protocol described, the speed of phosphorylation of the insecticides *k_p_* toward an enzyme can be founded from the values of the slope of inhibition, see Table 2.

The limit of detection (LOD) stated as ‘*A number expressed in units of concentration (or amount) that describes the lowest concentration level (or amount) of the element that an analyst can determine by the statistically different from an analytical blank*’ [29]. The LOD was determined according with [30] as follows: in the absence of inhibitor (steady-state current) the response of the biosensor was measured 10 times, the standard deviation σ was calculated, and the LOD was established at 3σ. The LOD values of each pesticide using the biosensor previously described are summarized in Table 2.

## 4. Conclusions

In the present work, a functional instrumentation for use in the detection of organophosphorus insecticides was presented. The complete system is integrated by a peristaltic pump, solenoid valves, a potentiostat, and a control algorithm based on LabVew^®^ implemented on a personal computer. Multi-commuted flow analysis (MCFA) has the advantage that every single element can be individually activated to perform parallel or sequential tasks.

The system presented can be susceptible to the noise due to the coupling of the potentiostat and the biosensor channels; this can be overcome with an improved isolated cable and with the implementation of digital filters. Nevertheless, the system presented in this work is a good approach for an amperometric flow analysis system that can be integrated in an electronic tongue to detect OP compounds in aqueous samples.

The sensor is inexpensive, small and used with the inhibition protocol proposed in the present work can detect smaller quantities than reported in similar prior works [18,33,34] due to the use of genetically-modified enzyme giving as a result higher sensitivity. This system offers the advantage of the ability to work with samples without any special pre-treatment, which increases the possibility of its use in *in situ* applications to carry out a large number of assays. The miniaturization of the flow cell is in progress. A smaller flow cell will allow faster measurements of enzymatic reactions. The use of an array of biosensors with different sensitivities and an analytical tool coupled with the detection system could further improve the use of the purposed system.

## Figures and Tables

**Table 1.** MCFA system repeatability obtained for two different speeds and four different volumes.

**Pump Speed (RPM)**	**Volume_set_**	**Time(seconds)**	**Volume_Mean_**	**RSD %**
**48**	100 μL	1	152.5 μL	4.96
	1 mL	10	0.99861 mL	1.42
	10 mL	60	9.9308 mL	1.48
**4.8**	100 μL	7	103.0 μL	1.58
	500 μL	35	529.5 μL	1.31
	1 mL	65	0.98269 mL	1.88

**Table 2.** Comparison between MCFA system described and previous FIA systems reported.

**Organophosphorus Insecticides**			
**Method**	**MCFA [this work]**		**FIA [31,32]**
Sampling	3 experiments/hour		---
Frequency			
Detection Limit (M)	CPO = 1.24 × 10^−9^, CFV= 1.262 × 10^−6^, AZMO = 1.48 × 10^−8^		---
R.S.D.%	1.58%		<5%
Pretreatment	Not required		Filtration
	**Phosphorylation constant (k_p_)**		
	mean	S.T.D.%	
CPO	0.258	0.922	----
CFV	0.166	7.208685	----
AZMO	0.0459	0.313976	----
